# Thromboembolic and bleeding complications during oral anticoagulation therapy in cancer patients with atrial fibrillation: a Danish nationwide population‐based cohort study

**DOI:** 10.1002/cam4.1054

**Published:** 2017-05-19

**Authors:** Anne G. Ording, Erzsébet Horváth‐Puhó, Kasper Adelborg, Lars Pedersen, Paolo Prandoni, Henrik T. Sørensen

**Affiliations:** ^1^ Department of Clinical EpidemiologyAarhus University HospitalAarhusDenmark; ^2^ Department of CardiologyAarhus University HospitalAarhusDenmark; ^3^ Department of Cardiovascular SciencesVascular Medicine UnitUniversity of PaduaPaduaItaly; ^4^ Department of Health Research and Policy (Epidemiology)Stanford Medical SchoolStanfordCalifornia

**Keywords:** Atrial fibrillation, atrial flutter, cohort, drug utilization, epidemiology, neoplasms

## Abstract

Coexisting cancer in patients with atrial fibrillation (AF) has been associated with thromboembolism and bleeding. We used Danish population‐based medical databases to conduct a population‐based cohort study that included all AF patients who redeemed a prescription for vitamin K antagonists (VKA) or non‐VKA oral anticoagulant (NOAC) between July 2004 and December 2013. We characterized these patients according to the presence (*N* = 11,855) or absence (*N* = 56,264) of a cancer diagnosis before redemption of their oral anticoagulant prescription, and then examined their 1‐year risk of thromboembolic or bleeding complications or death. We next used Cox regression to compare the hazard ratios for complications among VKA‐ or NOAC‐treated AF patients with versus without a cancer diagnosis, after adjusting for sex, age, and CHA_2_ DS_2_ VASc score. One‐year risks of thromboembolic complications in AF patients who redeemed a VKA prescription were similar in those with (6.5%) and without (5.8%) cancer [hazard ratio (HR) 1.0 (95% confidence interval (CI): 0.93, 1.1)]. This also was found for bleeding complications (5.4% vs. 4.3%, HR 1.1 [95% CI: 1.0, 1.2]). For AF patients with cancer who redeemed a NOAC prescription, risks were also similar for thromboembolic complications (4.9% of cancer patients vs. 5.1% of noncancer patients, HR 0.80 [95% CI: 0.61, 1.1]), and for bleeding complications (4.4% vs. 3.1%, HR 1.2 [95% CI: 0.92, 1.7]). The absolute risks of thromboembolic or bleeding complications were nearly the same in patients with and without cancer who redeemed prescription for VKAs or NOACs.

## Background

Cancer is a risk factor for atrial fibrillation (AF) 1, which predisposes patients to serious complications, such as a fivefold increased risk of stroke, a threefold increased risk of heart failure, and nearly a doubled risk of death 2, 3, 4. While oral anticoagulants are effective in reducing risk of stroke 4, 5, they increase risk of bleeding 6.

Since many cancers interact with the coagulation system, cancer patients are at particularly high risk of thromboembolic events, and bleeding 7, 8. Risk of recurrent venous thromboembolism (VTE) was found to be threefold higher in cancer patients after an initial VTE, compared to noncancer patients 9. When used to prevent VTE, vitamin K antagonists (VKAs) may increase the risk of bleeding up to sixfold in patients with cancer, compared with cancer‐free persons 6. In addition, response to VKA therapy can be affected by cancer drugs or coexisting conditions 10.

For cancer patients with AF, few data are available on adverse effects of anticoagulation. Although NOAC trials among AF patients included only a few cancer patients (and in particular those with assumed long life expectancy) 11, 12, 13, NOACs are prescribed increasingly to AF patients with cancer 7, 14.

Since both AF and cancer are common, any increased risk of thromboembolic events and bleeding may have major public health implications. We therefore examined the risks of thromboembolic and bleeding complications in AF patients with and without cancer who were prescribed a VKA or a NOAC.

## Methods

The Danish health care system provides free tax‐supported health care to the entire national population (cumulative population =  6,571,879 people during the study's 2004–2013 enrollment period). Nationwide registries within the health care system track diagnoses and procedures on the date of service, prescriptions on the date of redemption, and vital status for the entire population. Individual‐level data from these registries can be linked unambiguously using the unique civil personal registration (CPR) number assigned to all Danish residents at birth or upon immigration 15.

### Source population and data collection

The Danish National Patient Registry (DNPR) has recorded all inpatient hospitalizations in Denmark since 1977 and all outpatient and emergency room visits since 1995, using the *International Classification of Diseases*,* Eighth Revision* (IDC‐8) through 1993 and *Tenth Revision* (ICD‐10) thereafter 16. We used the DNPR to establish a cohort of all patients with any first‐time inpatient or hospital outpatient diagnosis of AF recorded between 1 January 2004 and 31 December 2013. We linked this cohort to the Danish National Health Service Prescription Database (DNHSPD), which has recorded information on redemption of reimbursed prescriptions from outpatient pharmacies since 2004 17. We then restricted the cohort to patients with first‐time AF who had at least one reimbursed prescription for a VKA or a NOAC within 90 days after their AF diagnosis. The cohort therefore included patients with first‐time AF, but not necessarily a first‐time prescription for VKA or NOAC. The index date was defined as the redemption date of the first reimbursed prescription. In a sensitivity analysis, we defined new users of VKA or NOAC as those with no previous record of VKA or NOAC prescription, respectively.

To identify all cancer diagnoses, we linked the AF cohort to the Danish Cancer Registry, which has recorded all incident cancers in Denmark since 1943 using ICD‐10 codes 18. We then divided the study cohort into patients with a previous diagnosis of incident cancer and those with no record of cancer as of the date of AF. Cancers were classified as gastrointestinal cancers, cancers of the lung or pleura, breast cancer, urological cancers, intracranial cancers, hematological cancers, and other cancers.

We used the DNPR to ascertain the medical history of all patients prior to their index date. We extracted information on diagnoses of cardiovascular comorbidities, obesity, thyroid diseases, chronic obstructive pulmonary disease, alcoholism, liver disease, and renal failure, as shown in Table 1. In addition, for each patient we calculated a CHA_2_DS_2_ VASc score 19, which is a risk prediction score for stroke in AF patients (Table S1). We extracted information on reimbursed prescriptions for cardiovascular comedications from the DNHSPD. Users were defined as persons with a record of at least one prescription for a given drug within 90 days before their index date. Variable definitions and diagnostic codes are provided in Table S1.

**Table 1:** Characteristics of atrial fibrillation patients with and without cancer who redeemed prescriptions for vitamin K antagonist or non‐vitamin K antagonist oral anticoagulants, Denmark, 1 July 2004–31 December 2013

	Vitamin K antagonists		Non‐vitamin K antagonist oral anticoagulants	
	No cancer (*n* = 49,057)	Cancer (*n* = 10,046)	No cancer (*n* = 7207)	Cancer (*n* = 1809)
Sex				
Female	19,399 (40)	4509 (45)	3186 (44)	923 (51)
Male	29,658 (60)	5537 (55)	4021 (56)	886 (49)
Median age (IQR)	72 (64, 79)	77 (70, 83)	72 (65, 80)	77 (70, 84)
Age group				
<65 years	13,431 (27)	1024 (10)	1707 (24)	168 (9.3)
65–74 years	16,299 (32)	3086 (31)	2521 (35)	580 (32)
75–79 years	8439 (17)	2250 (22)	1117 (16)	336 (19)
>=80 years	10,888 (22)	3686 (37)	1862 (26)	725 (40)
Cancer site				
Gastrointestinal cancer		1228 (12)		190 (11)
Cancer of the lung or pleura		404 (4.0)		56 (3.1)
Breast cancer		1192 (12)		224 (12)
Urological cancer		1468 (15)		248 (14)
Intracranial cancer		10 (0.1)		3 (0.2)
Hematological cancer		338 (3.4)		66 (3.6)
All other cancer sites		5406 (54)		1022 (56)
Cancer stage				
Localized		6685 (67)		1215 (67)
Regional		917 (9.1)		169 (9.3)
Distant		278 (2.8)		53 (2.9)
Unknown		2166 (22)		372 (21)
Time from last cancer diagnosis to index date				
0–2 years		2659 (27)		378 (21)
>2–5 years		2224 (22)		435 (24)
>5 years		5163 (51)		996 (55)
Index year				
2004–2007	18,218 (37)	3069 (31)	NA	NA
2008–2010	15,559 (32)	3156 (31)	9 (0.1)	4 (0.2)
2011–2013	15,280 (31)	3821 (38)	7198 (100)	1805 (100)
CHA_2_DS_2_ VASc score				
0	5960 (12)	378 (3.8)	745 (10)	65 (3.6)
1	6059 (12)	773 (7.7)	842 (12)	122 (6.7)
2	10,215 (21)	1978 (20)	1587 (22)	375 (21)
3	10,540 (22)	2599 (26)	1541 (21)	448 (25)
4	8341 (17)	2192 (22)	1272 (18)	407 (23)
5	4476 (9)	1195 (12)	704 (10)	208 (11)
6+	3466 (7)	931 (9.3)	516 (7.2)	184 (10)
Cardiovascular comorbidities				
Ischemic stroke	6190 (13)	1342 (13)	1093 (15)	296 (16)
Ischemic heart disease	11,016 (23)	2455 (24)	1365 (19)	337 (19)
Acute myocardial infarction	5265 (11)	1118 (11)	645 (8.9)	149 (8.0)
Congestive heart failure	4081 (8.3)	901 (9.0)	304 (4.2)	95 (5.3)
Valvular disease	5551 (11)	1207 (12)	411 (5.7)	117 (6.5)
Cardiomyopathy	21 (0)	5 (0)	*n* < 3	*N* < 3
Hypertension	27,723 (57)	6012 (60)	4177 (58)	1124 (62)
VTE	3330 (6.8)	962 (9.6)	279 (3.9)	101 (5.6)
Other comorbidities				
Diabetes	7293 (15)	1434 (14)	1123 (16)	244 (14)
Obesity	3218 (6.6)	563 (5.6)	493 (6.8)	99 (5.5)
Thyroid disease	1696 (3.5)	337 (3.4)	226 (3.1)	63 (3.5)
Chronic obstructive pulmonary disease	12,353 (25)	2802 (28)	1969 (27)	558 (31)
Alcoholism	1660 (3.4)	256 (2.5)	310 (4.3)	49 (2.7)
Liver disease	414 (0.8)	77 (0.8)	60 (0.8)	12 (0.7)
Renal failure	1720 (3.5)	474 (4.7)	128 (1.8)	46 (2.5)
Cardiovascular comedication^a^				
Statins	11,835 (24)	2382 (24)	1993 (28)	452 (25)
Acetylsalicylic acid	13,206 (27)	2786 (28)	1603 (22)	437 (24)
Non‐aspirin NSAIDs	5034 (10)	1019 (10)	721 (10)	169 (9.3)
ACE or A2R inhibitors	16,879 (34)	3404 (34)	2555 (36)	626 (35)
Calcium channel blockers	9849 (20)	2200 (22)	1499 (21)	394 (22)
Beta‐blockers	18,567 (38)	3728 (37)	2516 (35)	651 (36)
Digoxin	6295 (13)	1478 (15)	474 (6.6)	168 (9.3)
Diuretics	16,500 (34)	3746 (37)	1945 (27)	537 (30)
Platelet inhibitors (dipyrammol, ticagrelor, clopidogrel)	2913 (5.9)	702 (7.0)	553 (7.7)	147 (8.1)
Amiodaron	873 (1.8)	153 (1,5)	55 (0.8)	14 (0.8)
New users of oral anticoagulation^b^	33,642 (69)	6625 (66)	5999 (83)	1500 (83)

*n* (%), unless otherwise specified. VTE, venous thromboembolism.

a Comedication defined as at least one reimbursed prescription recorded within 90 days of the index prescription for a VKA or NOAC.

b New users defined as patients with no history of a prescription for oral anticoagulation in the registry (with at least 6 months of prescription history).

### Follow‐up

The study outcome was time from the index date to a thromboembolic complication (defined as any inpatient or outpatient diagnosis of ischemic stroke, VTE, other arterial embolism, or myocardial infarction) or to a bleeding complication (defined as any inpatient or hospital outpatient diagnosis of hemorrhagic stroke or gastrointestinal, lung, or urinary hemorrhage) recorded in the DNPR 16. Using the Civil Registration System, we followed patients for 1 year, or until death, emigration, or 31 December 2013, whichever came first 15.

### Statistical analysis

**Figure 1:**
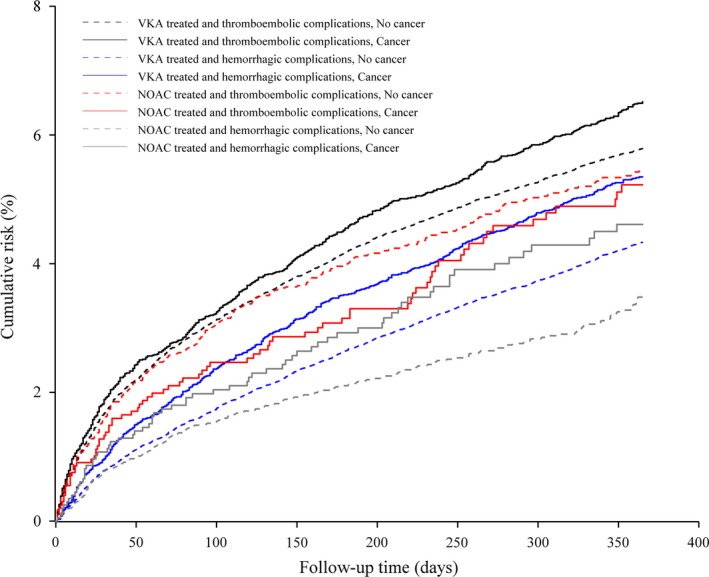
Cumulative risk of thromboembolic complications and bleeding complications in atrial fibrillation patients with and without a previous cancer diagnosis who used vitamin K antagonists (VKA) or non‐vitamin K antagonist oral anticoagulants (NOAC), Denmark, July 2004–December 2013.

We tabulated frequencies of all baseline covariates in the cancer and noncancer groups (Table 1). We then used cumulative incidence functions to compute 1‐year risks for thromboembolic or bleeding complications among cancer and noncancer patients who had redeemed prescriptions for a VKA or a NOAC, accounting for death as a competing risk (Figure 1) 20. Risks were calculated overall and by categories defined by covariates.

We used Cox regression to compute hazard ratios (HRs) comparing outcomes in patients with cancer to outcomes in those without cancer, adjusting for sex, age group (<65 years, 65–74 years, 75–79 years, and ≥80 years), and CHA_2_DS_2_ VASc score (0, 1, 2, 3, 4, 5, and ≥6). We computed hazard rate ratios separately for patients prescribed a VKA versus a NOAC. Due to low numbers of patients, we then compared all cancer patients with redeemed VKA or NOAC prescriptions in categories defined by time since the most recent cancer diagnosis (<2 years, 2‐<5 years, and ≥5 years) and by cancer site to all noncancer patients. The proportionality assumption was assessed graphically using log minus log plots and found to be appropriate.

To account for the excess risk of a subsequent event associated with a previous thromboembolic or bleeding episode, we conducted a sensitivity analysis in which we excluded patients (*n *=* *23,206) who had a thromboembolic or bleeding diagnosis before the index date. We also conducted a sensitivity analysis that included only patients defined as new users of oral anticoagulation therapy. In a posthoc analysis, we restricted the Cox regression models to patients with index date in the calendar years 2011 through 2013, to examine the risks of thromboembolic and bleeding events among patients in the period where NOAC was available.

We also conducted a posthoc sensitivity analysis restricting to patients with at least two prescriptions for VKA or NOAC, respectively, during follow‐up. In this analysis, patients were followed from their second reimbursed prescription.

Analyses were conducted using SAS version 9.4 (SAS Institute, Cary, NC). The study was approved by the Danish Data Protection Agency (record number 2012‐41‐0793).

## Results

Descriptive characteristics of the study population are presented in Table 1. Among AF patients with VKA prescriptions, we identified 49,057 (83%) noncancer and 10,046 (17%) cancer patients. Among those with redeemed NOAC prescriptions, 7207 (80%) were noncancer patients and 1809 (20%) were cancer patients. Patients with redeemed VKA prescriptions were followed for a total of 51,506 years for thromboembolic complications and 52,121 years for bleeding complications, while patients with redeemed NOAC prescriptions were followed for a total of 5740 years for thromboembolic complications and 5819 years for bleeding complications.

Noncancer patients were younger than cancer patients (median age 72 years (IQR: 64, 79 years) and 77 years (IQR: 70, 83 years), respectively, and 54% of cancer patients were male compared with 60% of noncancer patients. Comorbidity was frequent, particularly hypertension, which was diagnosed in 57% of noncancer patients and 60% of cancer patients. Urological cancer was diagnosed in 15% of cancer patients, followed by breast cancer (12%), gastrointestinal cancer (12%), lung cancer (4%), hematological cancer (3%), and intracranial cancer (0.1%). Other sites accounted for 54% of cancers. More than half (52%) of cancers were diagnosed for five or more years before the index date.

### Risk of thromboembolic and bleeding complications

One‐year risks of thromboembolic complications in AF patients who redeemed a VKA prescription were similar in those with (6.5%) and without (5.8%) cancer (hazard ratio [HR] 1.0 [95% confidence interval [CI]: 0.93, 1.1]). This also was found for bleeding complications (5.4% vs. 4.2%, HR 1.1 [95% CI: 1.0, 1.2]). For AF patients with cancer who redeemed a NOAC prescription, risks were also similar for thromboembolic complications (4.9% of cancer patients vs. 5.1% of noncancer patients, HR 0.80 [95% CI: 0.61, 1.1]), and for bleeding complications (4.4% vs. 3.1%, HR 1.2 [95% CI: 0.92, 1.7]). In the posthoc analysis restricted to patients with index dates in 2011–2013, the results were nearly similar. The adjusted HR for thromboembolic events was 1.1 (95% CI: 0.91, 1.2) for patients with VKA prescription, and 0.82 (95% CI: 0.62, 1.1) for patients with NOAC prescription. Similar results were found for bleeding complications; 1.0 (95% CI: 0.87, 1.2) for VKA prescription and 1.2 (95% CI: 0.90, 1.7) for NOAC prescription.

When restricting to patients with at least two prescriptions for the same anticoagulant drug, the median time between first and second prescription was 52 days (IQR: 37–76) for VKA prescriptions and 29 days (IQR: 22–44) for NOAC prescriptions. In this analysis, we also found similar HRs for thromboembolic and bleeding events as in the main analysis: 1.0 (95% CI: 0.92, 1.2) for thromboembolic events after two VKA prescriptions and 0.98 (95% CI: 0.71, 1.4) for NOAC prescriptions. The HRs for bleeding complications were 1.1 (95% CI: 0.0.95, 1.2) and 1.2 (95% CI: 0.85, 1.7), respectively.

For patients with VKA prescription, one‐year risks were higher for ischemic stroke in all patients who redeemed prescriptions for anticoagulants, but those who redeemed VKA prescriptions were also at risk of subsequent VTE (1.7% of cancer patients and 1.1% of noncancer patients). In contrast, patients with NOAC prescriptions were at risk of myocardial infarction (1.1% of cancer patients and 1.2% of noncancer patients). Lung and urinary bleeding were the most common bleeding complications. These risks were slightly lower for noncancer patients than for cancer patients. (Table 2)

**Table 2:** Absolute risk in percent and adjusted hazard ratios (HRs) with 95% confidence intervals (CI) of thromboembolic and bleeding complications in patients with atrial fibrillation during the first year after a prescription for a vitamin K antagonist or a non‐vitamin K antagonist oral anticoagulant in patients with and without cancer, Denmark, July 2004 through December 2013

*Vitamin K antagonist*	No cancer		Cancer		HR (95% CI)^a^	HR (95% CI)^b^
	Events, *n*	Risk (95% CI)	Events, *n*	Risk (95% CI)		
Thromboembolic complications^c^	2734	5.8 (5.6, 6.0)	628	6.5 (6.0, 7.0)	1.0 (0.93, 1.1)	1.1 (0.91, 1.2)
Ischemic stroke	1426	3.1 (3.0 3.3)	302	3.4 (3.0, 3.7)	0.92 (0.81, 1.1)	0.92 (0.73, 1.2)
Venous thromboembolism	527	1.1 (1.0, 1.2)	162	1.7 (1.5, 2.0)	1.4 (1.2, 1.7)	1.7 (1.2, 2.5)
Arterial embolism	70	0.15 (0.12, 0.19)	18	0.19 (0.12, 0.30)	1.1 (0.66, 1.9)	1.2 (0.53, 2.9)
Myocardial infarction	739	1.6 (1.5, 1.7)	154	1.6 (1.4, 1.9)	0.93 (0.78, 1.1)	1.0 (0.75, 1.3)
Bleeding complications^c^	2025	4.2 (4.1, 4.5)	513	5.4 (4.9, 5.8)	1.1 (1.0, 1.2)	1.2 (1.0, 1.3)
Hemorrhagic stroke	229	0.49 (0.43, 0.56)	52	0.54 (0.41, 0.71)	0.94 (0.70, 1.3)	1.2 (0.83, 1.8)
Gastrointestinal hemorrhage	690	1.5 (1.4, 1.6)	184	1.9 (1.7, 2.2)	1.1 (0.95, 1.3)	1.2 (0.94, 1.5)
Lung and urinary hemorrhage	1111	2.4 (2.3, 2.5)	277	2.9 (2.6, 3.2)	1.2 (1.0, 1.3)	1.1 (0.92, 1.4)
Non‐vitamin K antagonist oral anticoagulant						
Thromboembolic complications^c^	290	5.1 (4.5, 5.7)	65	4.9 (3.8, 6.2)	0.80 (0.61, 1.1)	1.2 (0.78, 1.9)
Ischemic stroke	188	3.4 (3.0, 4.0)	40	3.0 (2.2, 4.1)	0.77 (0.55, 1.1)	1.2 (0.67, 2.2)
Venous thromboembolism	30	0.53 (0.36, 0.75)	12	0.98 (0.53, 1.7)	1.4 (0.72, 2.8)	3.9 (1.3, 11)
Arterial embolism	8	0.17 (0.08, 0.33)	0	N/A	N/A	N/A
Acute myocardial infarction	65	1.2 (0.91, 1.5)	13	1.1 (0.61, 1.8)	0.72 (0.39, 1.3)	0.73 (0.25, 2.2)
Bleeding complications^c^	166	3.1 (2.6, 3.6)	60	4.4 (3.4, 5.6)	1.2 (0.92, 1.7)	1.2 (0.74, 1.8)
Hemorrhagic stroke	10	0.21 (0.11, 0.39)	6	0.56 (0.23, 1.2)	2.2 (0.77, 6.1)	2.5 (0.78, 7.8)
Gastrointestinal hemorrhage	53	0.92 (0.70, 1.2)	27	1.8 (1.2, 2.6)	1.7 (1.0, 2.7)	1.5 (0.77, 3.0)
Lung and urinary hemorrhage	103	2.0 (1.6, 2.4)	27	2.0 (1.4, 2.9)	0.92 (0.60, 1.4)	0.75 (0.38, 1.5)

N/A indicates no cases.

a Hazard ratios comparing thromboembolic and bleeding complications and death in atrial fibrillation patients with cancer versus without cancer.

b Excludes patients (*n* = 23,206) with prevalent thromboembolic or bleeding events.

c Events were not mutually exclusive. The number of specific events therefore does not necessarily add up to the total number of events.

After excluding patients with thromboembolic or bleeding events prior to the index date (*n *=* *23,206), the HR of thromboembolic complications among remaining cancer patients was similar to that for all cancer patients for VKA prescriptions, but increased from 0.80 in the full cancer patient cohort to 1.2 after exclusions for NOAC prescriptions. HRs were increased particularly for VTE: from 1.4 (95% CI: 1.2, 1.7) to 1.7 (95% CI: 1.2, 2.5) for VKA prescriptions and from 1.4 (95% CI: 0.72, 2.8) to 3.9 (95% CI: 1.3, 11) for NOAC prescriptions. Overall HRs for bleeding complications were similar after excluding patients with previous events, but the relative risk for hemorrhagic stroke increased from 0.94 to 1.2 among patients with VKA prescriptions and from 2.2 to 2.5 for patients with NOAC prescriptions (Table 2).

HRs was very similar when the analysis was restricted to patients defined as new users of VKAs or NOACs (data not shown).

When we combined the cohorts of patients with VKA or NOAC prescriptions and examined patients with and without cancer, active cancer (i.e., diagnosed <2 years before the index date) was slightly associated with an increased relative risk of thromboembolic or bleeding events among cancer patients compared with noncancer patients. Patients with cancer of the lung or pleura (HR 2.0, 95% CI: 1.4, 2.8) or with urological cancer (HR 1.7, 95% CI: 1.4, 2.0) were at increased risk of bleeding events. Risks of other bleeding events were imprecisely measured (Table 3).

**Table 3:** Hazard ratios (HRs) and 95% confidence interval (CIs) comparing thromboembolic and bleeding complications in the first year after a vitamin K antagonist or non‐vitamin K antagonist oral anticoagulant prescription among AF patients with vs. without cancer by time since last cancer diagnosis and cancer type, Denmark, July 2004 through December 2013

	Thromboembolic complications	Bleeding complications
	HR (95% CI)	HR (95% CI)
Time since cancer diagnosis		
<2 years	1.1 (0.99, 1.3)	1.2 (1.0, 1.4)
2 to <5 years	0.92 (0.78, 1.1)	1.1 (0.93, 1.3)
≥5 years	0.95 (0.85, 1.1)	1.1 (0.95, 1.2)
Cancer site		
Gastrointestinal cancer	1.2 (0.94, 1.4)	1.1 (0.85, 1.4)
Cancer of the lung or pleura	1.5 (1.1, 2.2)	2.0 (1.4, 2.8)
Breast cancer	0.78 (0.61, 0.99)	0.85 (0.63, 1.2)
Urological cancer	1.0 (0.83, 1.3)	1.7 (1.4, 2.0)
Intracranial cancer	2.2 (0.31,16)	N/A
Hematological cancer	0.65 (0.38, 1.1)	0.61 (0.33, 1.1)
All other cancer sites	0.99 (0.89, 1.1)	1.0 (0.89, 1.2)

Hazard ratios comparing patients with cancer to patients without cancer and adjusting for sex, age group, and CHA_2_DS_2_ VASc score.

N/A indicates no cases.

Specific cumulative risks stratified by sex, age group, cancer stage, and CHA2DS‐VASc score are presented in Table S2.

## Discussion

Risks of thromboembolic and bleeding events were found to be almost the same in AF patients with and without prevalent cancer, irrespective of whether they were prescribed VKAs or NOACs. As expected, compared to noncancer patients, cancer patients were at higher risk for VTE and for lung and urinary bleeding.

This study was based on a nationwide cohort of patients with AF treated in a tax‐supported and uniformly organized health care system with complete follow‐up. Limitations included lack of information on dosage of anticoagulation, whether patients complied with treatment or switched to another drug during follow‐up, on physician preference for type of drug. However, the majority of patients were new users of VKAs (68%) or NOACs (83%) and only 4% of patients switched drugs during follow‐up, and restricting analyses to those with at least two prescriptions for the same anticoagulant did not change the estimate of associations. Another concern is that we lacked information on clinical and lifestyle factors that constitute risk factors for thromboembolic or bleeding conditions, such as smoking, body weight, and HAS‐BLED score. Still, we did have information on comorbidities such as diabetes, alcoholism, chronic pulmonary disease, and other lifestyle‐related conditions, and found that they were similarly distributed between the noncancer and cancer cohorts. Thus, there is no indication that such factors explain our findings.

While three studies have compared the risk of recurrent thromboembolism in cancer and noncancer patients 6, 21, 22, thus far none have specifically quantified the risk of thromboembolic or bleeding complications in cancer and noncancer patients with AF according to use of VKAs or NOACs. Existing guidelines for VTE prevention in cancer patients still recommend use of low molecular weight heparin (LMWH) for primary prophylaxis and LMWH/VKA for initial and long‐term treatment 14, 23, 24, 25. Previous clinical trials of cancer patients treated with VKAs or NOACs for VTE suggest that NOACs may be effective and safe in selected patients with active cancer 14, 26. Unfortunately, our cohort of cancer patients was too small to examine time since cancer diagnosis as a predictor of complications in NOAC‐treated patients.

Anticoagulant therapy may be difficult to manage in patients with active cancer 14, 22, 27, and the physician choice for oral anticoagulant therapy may depend on type of cancer and time since cancer diagnosis. Patients with active cancer treated with VKA therapy may not meet the desired international normalized ratio (INR) interval due to hypercoagulation, interaction with oncology drugs, or concurrent conditions 6, 8, 10, 28, but the frequencies of comorbidities did not vary substantial among cancer patients with VKA versus NOAC prescriptions in our study. At the same time, NOAC therapy may interact with several classes of drugs through common metabolic pathways such as CYP3A4 14.

Our results indicated a slightly increased risk associated with active cancer, but we lacked information on hospital‐based anticoagulant prophylaxis and thus may not have captured all patients with newly diagnosed cancer. VTE is a frequent complication of cancer, supporting our finding of a higher risk for VTE than for other thromboembolic events among cancer patients. It is well known that patients with cancer and a previous thromboembolic event have at least a twofold increased risk of a new event, compared to similar patients with no malignancy 6, 21, 29. After excluding patients with previous thromboembolic or bleeding events, the overall relative risk of such complications was similar in cancer and noncancer patients with VKA prescriptions, but slightly increased for thromboembolic events among cancer patients with NOAC prescriptions. We speculate that patients with previous events are current and well‐controlled users of VKA therapy and that the remaining group of patients more often represented new users. Indeed, 67% of excluded patients with a VKA prescription and 85% of patients with a NOAC prescription were defined as new users.

In conclusion, our study found similar risks of thromboembolic or bleeding events in AF patients with and without cancer irrespective of VKA or NOAC prescription.

## Conflict of Interest

The authors declare no conflicts of interest.

## Supporting information


**Table S1.** Variables and definitions.
**Table S2.** Absolute risks in percent with 95% confidence intervals of thromboembolic and bleeding complications in patients with atrial fibrillation during the first year after redeeming a prescription for a vitamin K antagonist or a non‐vitamin K antagonist oral anticoagulant in patients with and without cancer, Denmark, July 2004 ‐ December 2013.
