# Supplementary material for: Thromboembolic and bleeding complications during oral anticoagulation therapy in cancer patients with atrial fibrillation: a Danish nationwide population‐based cohort study
Source: Cancer Med. 2017 May 19;6(6):1165–72. doi: 10.1002/cam4.1054 (PMC5463075; doi:10.1002/cam4.1054)
Supplement: Supplementary file 1 — Table S1. Variables and definitions. Table S2. Absolute risks in percent with 95% confidence intervals of thromboembolic and bleeding complications in patients with atrial fibrillation during the first year after redeeming a prescription for a vitamin K antagonist or a non‐vitamin K antagonist oral anticoagulant in patients with and without cancer, Denmark, July 2004 ‐ December 2013. [file CAM4-6-1165-s001.docx]

**Supporting information**

**Supporting Information Table S1.** Variables and definitions.
 **Supporting Information Table S2.** Absolute risks in percent with 95% confidence intervals of thromboembolic and bleeding complications in patients with atrial fibrillation during the first year after redeeming a prescription for a vitamin K antagonist or a non-vitamin K antagonist oral anticoagulant in patients with and without cancer, Denmark, July 2004 - December 2013.

| **Supporting Information Table S1. Variables and definitions.** | |
| --- | --- |
| **Atrial fibrillation or atrial flutter** | ICD-8: 42793, 42794, ICD-10: I48 |
| **Cancer** | ICD-10: C00-C99 |
| **NOAC (dabigatran etexilat, rivaroxaban, apixaban)** | ATC: B01AE07, B01AF01, B01AF02 |
| **Vitamin K antagonists** | ATC: B01AA03, B01AA04 |
| **Gastrointestinal cancer** | ICD-10: C15-C26 |
| **Lung cancer incl. pleura** | ICD-10: C33-C34, C384, C450 |
| **Breast cancer** | ICD-10: C50 |
| **Urological cancer** | ICD-10: C61, C64-C68 |
| **Intracranial cancer** | ICD-10: C71, C751–C753 |
| **Hematological cancer** | ICD-10: C91-C96, C81-C86, C88 |
| **Ischemic stroke** | ICD-8: 433-434, ICD-10 I63-I64 |
| **Hemorrhagic stroke** | ICD-8: 430-431, ICD-10 I60,I61 |
| **Gastrointestinal hemorrhage** | ICD-10: K25.0, K25.2, K25.4, K25.6, K26.0, K26.2, K26.4, K26.6, K27.0, K27.2, K27.4, K27.6, K28.0, K28.2, K28.4, K28.6, K29.0, and K92.0-K92.2 |
| **Lung and urinary hemorrhage** | ICD-10: J942, N02, R04, R31 |
| **VTE** | ICD-8:451, 450.99, ICD-10: I80.1-3, I26 |
| **Arterial embolism** | ICD-8: 444, ICD-10: I74 |
| **Acute myocardial infarction** | ICD-8: 410, ICD-10: I21 |
| **Calcium channel blockers** | ATC: C08 |
| **Beta-blockers** | ATC: C07 |
| **Digoxin** | ATC: C01AA05 |
| **Statins** | ATC: C10AA (except C01AA05), C10B, B04AB |
| **Acetylsalicylic acid** | ATC: B01AC06, N02BA01 |
| **Non-aspirin NSAIDs** | ATC: M01A, except M01AX05 |
| **Clopidogrel** | ATC: B01AC04 |
| **Diuretics** | ATC: C03 |
| **Amiodaron** | ATC: C01BD01 |
| **Platelet inhibitors (ticagrelor, dipyrammol)** | ATC: B01AC07, B01AC24 |
| **CHA_2_DS_2_ VASc score** | Sum of weights for each patient:  Female sex (weight=1)  **Age 65-74 years (weight=1)**  **Age ≥ 75 years (weight=2)**  **Diabetes mellitus (weight=1)**  **Stroke/TIA/peripheral embolism (weight=2)** Vascular disease (Myocardial infarct, peripheral arterial disease) (weight=1)  Congestive heart failure (weight=1)  Hypertension (weight=1) |
| **Variables included in the CHA_2_DS_2_ VASc-score** | Heart failure: ICD-8: 425, 4270, 4271, ICD-10: I110, I42, I50, J819 and at least one prescription for loop diuretics redeemed within 180 days prior to index date, ATC: C03C  Hypertension: ICD-8: 400-404, ICD-10: I10-I15, I67.4. Combination treatment of at least two redeemed prescriptions for different types of the following classes of antihypertensive drugs within 180 days prior to index date: α adrenergic blockers ATC: C02A, C02B, C02C, non-loop diuretics ATC: C02DA, C02L, C03A, C03B, C03D, C03E, C03X, C07C, C07D, C08G, C09BA, C09DA, C09XA52, vasodilators, ATC: C02DB, C02DD, C02DG, C04, C05, β-blockers, ATC: C07, calcium channel blockers, ATC: C07F, C08, C09BB, C09DB, and renin-angiotensin system inhibitors ATC: C09  Diabetes mellitus: ICD-8: 249, 250. ICD-10: E10-14, H36.0. A claimed prescription for a glucose lowering drug within 180 days prior to index date, ATC: A10  **Stroke/TIA/peripheral embolism (**as defined in ^19^**, includes peripheral artery embolism, stroke, transient ischemic attack and pulmonary embolism): ICD-8: 433-438, 444, 450, ICD-10: )G458, G459, I26, I63, I64, I74).**  Vascular disease (as defined in ^19^**, includes myocardial infarction, peripheral artery disease, aortic plaque) ICD-8: 410, 440, ICD-10: I21, I22, I700, I702–I709** |

| **Supporting Information Table S2. Absolute risks in percent (with 95% confidence intervals) of thromboembolic and bleeding complications in patients with atrial fibrillation during the first year after redeeming a prescription for a vitamin K antagonist or a non-vitamin K antagonist oral anticoagulant among patients with and without cancer, Denmark, July 2004 – 31 December 2013.** | | | | | | | | | |
| --- | --- | --- | --- | --- | --- | --- | --- | --- | --- |
|  | **Vitamin K antagonists** | | | |  | **Non-vitamin K antagonist oral anticoagulants** | | | |
|  | **Thromboembolic complications** | | **Bleeding complications** | |  | **Thromboembolic complications** | | **Bleeding complications** | |
|  | No cancer | Cancer | No cancer | Cancer |  | No cancer | Cancer | No cancer | Cancer |
| **Sex** |  |  |  |  |  |  |  |  |  |
| Male | 5.4 (5.1, 5.6) | 6.3 (5.7, 7.0) | 4.5 (4.3, 4.8) | 6.8 (6.2, 7.5) |  | 5.0 (4.3, 5.8) | 5.7 (4.0, 7.7) | 3.7 (3.0, 4.4) | 5.0 (3.5, 6.9) |
| Female | 6.5 (6.1, 6.8) | 6.8 (6.0, 7.5) | 4.0 (3.8, 4.3) | 3.6 (3.0, 4.1) |  | 5.3 (4.4 6.2) | 4.2 (2.8, 6.0) | 2.4 (1.8, 3.1) | 3.8 (2.5, 5.5) |
| **Age group** |  |  |  |  |  |  |  |  |  |
| <65 years | 4.0 (3.7, 4.3) | 4.9 (3.7, 6.4) | 2.6 (2.4, 2.9) | 3.3 (2.3, 4.6) |  | 3.7 (2.7, 4.8) | 3.3 (1.1, 7.8) | 2.1 (1.4, 3.1) | 1.8 (0.32, 6.0) |
| 65-74 years | 5.4 (5.0, 5.7) | 5.1 (4.4, 6.0) | 4.2 (3.9, 4.5) | 4.8 (4.1, 5.6) |  | 4.1 (3.2, 5.0) | 2.7 (1.5, 4.7) | 2.7 (2.0, 3.5) | 1.8 (0.84, 3.5) |
| 75-79 years | 6.7 (6.1, 7.2) | 6.9 (5.8, 8.0) | 5.2 (4.7, 5.7) | 5.9 (4.9, 6.9) |  | 5.7 (4.2, 7.4) | 6.7 (4.0, 10) | 2.1 (1.3, 3.3) | 6.5 (3.8, 10) |
| >=80 years | 8.1 (7.6, 8.6) | 8.0 (7.1, 8.9) | 6.1 (5.6, 6.5) | 6.1 (5.3, 6.9) |  | 7.5 (6.2, 9.0) | 6.2 (4.3, 8.6) | 5.2 (4.1, 6.5) | 6.1 (4.3, 8.4) |
| **Cancer stage** |  |  |  |  |  |  |  |  |  |
| Localized | N/A | 6.1 (5.5, 6.7) | N/A | 5.3 (4.8, 5.9) |  | N/A | 4.8 (3.5, 6.4) | N/A | 3.9 (2.7, 5.3) |
| Regional | N/A | 8.2 (6.5, 10) | N/A | 4.0 (2.8, 5.4) |  | N/A | 5.2 (2.1, 10) | N/A | 3.8 (1.4, 8.1) |
| Distant | N/A | 9.7 (6.5, 14) | N/A | 5.7 (3.3, 8.9) |  | N/A | 2.3 (0.18, 10) | N/A | 9.5 (1.9, 2.4) |
| Unknown | N/A | 6.8 (5.8, 7.9) | N/A | 5.9 (5.0 7.0) |  | N/A | 5.3 (3.0, 8.5) | N/A | 5.8 (3.4, 9.2) |
| **CHA2DS2 VASc-score** |  |  |  |  |  |  |  |  |  |
| 0 | 2.2 (1.8, 2.6) | 4.1 (2.4, 6.5) | 1.7 (1.4, 2.0) | 2.4 (1.2, 4.4) |  | 2.1 (1.1, 3.6) | 4.6 (0.77, 14) | 1.4 (0.61,2.8) | 3.3 (0.25, 14) |
| 1 | 2.9 (2.5, 3.3) | 3.9 (2.7, 5.5) | 2.8 (2.4, 3.3) | 4.2 (2.9, 5.8) |  | 2.0 (1.2, 3.2) | 1.8 (0.34, 5.7) | 2.8 (1.6, 4.4) | 0.99 (0.09,4.9) |
| 2 | 4.1 (3.7, 4.5) | 4.4 (3.6, 5.4) | 4.1 (3.8, 4.6) | 4.2 (3.4, 5.2) |  | 3.3 (2.4, 4.5) | 4.0 (2.0, 7.2) | 2.6 (1.8, 3.6) | 1.9, (0.78, 4.0) |
| 3 | 5.5 (5.1, 6.0) | 5.3 (4.5, 6.2) | 4.8 (4.4, 5.2) | 5.9 (5.0, 6.9) |  | 4.8 (3.6, 6.2) | 4.1 (2.2, 6.8) | 3.0 (2.1, 4.1) | 4.8 (2.8, 7.5) |
| 4 | 6.6 (6.1, 7.2) | 6.1 (5.1, 7.1) | 5.3 (4.8, 5.8) | 6.1 (5.2, 7.2) |  | 5.9 (4.5, 7.6) | 2.9 (1.3, 5.5) | 3.7 (2.6, 5.0) | 7.1 (4.5, 10) |
| 5 | 11 (10, 12) | 9.1 (7.5, 11) | 6.1 (5.4, 6.8) | 5.8 (4.6, 7.3) |  | 9.0 (6.7, 12) | 12 (7.4, 18) | 4.8 (3.1, 6.9) | 4.8 (2.1, 9.3) |
| >=6 | 14 (13, 16) | 15 (13, 18) | 6.5 (5.7, 7.4) | 6.0 (4.5, 7.7) |  | 14 (11, 18) | 7.4 (3.9, 12) | 4.5 (2.6, 7.2) | 4.7 (2.0, 9.0) |
